# CircRNA hsa_circRNA_104348 promotes hepatocellular carcinoma progression through modulating miR-187-3p/RTKN2 axis and activating Wnt/β-catenin pathway

**DOI:** 10.1038/s41419-020-03276-1

**Published:** 2020-12-14

**Authors:** Guanqun Huang, Min Liang, Haiyan Liu, Jianhong Huang, Peiqing Li, Chong Wang, Yidan Zhang, Ye Lin, Xianhan Jiang

**Affiliations:** 1 Department of General Surgery, The Fifth Affiliated Hospital of Guangzhou Medical University, 510700 Guangzhou City, Guangdong Province China; 2 Phase I Clinical Trial Center, The Fifth Affiliated Hospital of Guangzhou Medical University, 510700 Guangzhou City, Guangdong Province China; 3 Department of General Surgery, Guangdong General Hospital, Guangdong Academy of Medical Sciences, 510080 Guangzhou City, Guangdong Province China; 4 Department of Abdominal Oncology, The Fifth Affiliated Hospital of Guangzhou Medical University, 510700 Guangzhou City, Guangdong Province China

**Keywords:** Biotechnology, Cancer

## Abstract

Circular RNAs (circRNAs) have confirmed to participate in diverse biological functions in cancer. However, the expression patterns of circRNAs on hepatocellular carcinoma (HCC) remains unclear. In the present study, we clarified that hsa_circRNA_104348 was dramatically upregulated in HCC tissues and cells. Patients with HCC displaying high hsa_circRNA_104348 level possessed poor prognosis. Has_circ_104348 facilitated proliferation, migration, and invasion, meanwhile suppressed apoptosis of HCC cell. Furthermore, hsa_circRNA_104348 directly targeted miR-187–3p, could regulate miR-187-3p to affect proliferation, migration, invasion, and apoptosis of HCC cells, and may have effect on Wnt/β-catenin signaling pathway. Moreover, RTKN2 could be a direct target of miR-187-3p. In addition, knockdown of hsa_circRNA_104348 attenuated HCC tumorigenesis and lung metastasis in vivo. Taken together, these findings indicated that circular RNA hsa_circRNA_104348 might function as a competing endogenous RNA (ceRNA) to promotes HCC progression by targeting miR-187–3p/RTKN2 axis and activating Wnt/β-catenin pathway.

## Introduction

Hepatocellular carcinoma (HCC) is known as a common primary liver cancer globally, with an increasing prevalence worldwide^1^. It is one of the main cause of cancer-related mortality across the world, especially in China^2^. Cirrhosis caused by chronic hepatitis B or hepatitis C is the main risk factor and accounts for the majority of HCC cases^3,4^. Other leading risk factors include immune-related hepatitis, diabetes, and nonalcoholic fatty liver disease^5^. Recent treatment strategies include local ablation, catheter-based locoregional treatment, curative resection, chemotherapy, immune checkpoint inhibitors, depending on tumor characteristics and different stages of HCC^6,7^. Despite the achievements have been made in this field, the prognosis of patients with HCC remain very poor due to the tumor recurrence and metastasis, drug resistance and high adverse side effects^7^. Therefore, novel bio-markers for HCC treatment, as well as their underling molecular mechanisms are urgently needed.

In recent years, circular RNAs (circRNAs) have attracted much attention. CircRNAs are known as a certain class of non-coding RNAs which produced by backsplicing^8^. Previously, they were known as ‘junk’ generated by aberrant splicing processes without any biological function^9^. However, accumulating evidence has confirmed that circRNAs were able to regulate gene expression at the transcriptional or post-transcriptional level by contacting with microRNAs (miRNAs) or other molecules^10,11^. CircRNAs are usually more stable and resistant to decay machineries than linear RNAs, due to the covalent closed-loop structure lack of 3′ and 5′ ends^12^. Abnormal expression of circRNAs occurs in vinous of cancers, e.g. gastric cancer^13^, breast cancer^14^, lung cancer^15^, colorectal cancer^16^, indicating that they may paly critical roles in carcinogenesis. In HCC, several circRNAs have been clarified to be involved in HCC progression, e.g. cSMARCA5^17^, circRHOT1^18^, circTRIM33-12^19^. Different from circRNAs’s structure, miRNAs are another cluster of small non-coding RNAs which have a length of ∼22 nucleotides^20^, which considered to be important mediators in many biological process involved in HCC progression^21^. Moreover, increasing researchers have focused on the interactions between circRNAs and miRNAs in HCC. For instance, Han et al.’s^22^ study found that circMTO1 could suppress proliferation and invasion of HCC cells, and may act as the sponge of oncogenic miR-9 to promote p21 expression. In addition, another circRNA, named hsa_circ_0091570, has confirmed to act as a ceRNA of hsa-miR-1307 to inhibit HCC progression. Although many studies, not limited to above studies, already presented many molecular mechanisms of circRNAs and miRNAs in HCC, further research are still needed to clarify the whole regulatory networks of circRNAs and their down-stream factors.

In our study, by analyzing human HCC tissue, we clarified a specific circRNA named hsa_circRNA_104348 significantly upregulated and closely related to the prognosis of patients with HCC. Thus, the aim of the current study was to clarify the role of hsa_circRNA_104348 in HCC progression and its potential underling mechanisms. Our findings might shed a novel light on pathophysiologic mechanism of HCC and may provide new therapeutic target for HCC treatment.

## Materials and methods

### HCC tissue collection

Tissue samples were obtained in The Fifth Affiliated Hospital of Guangzhou Medical University. A total number of 60 pairs of HCC tissue and adjacent normal tissues were included. All human-related procedures performed in our study were in accordance with the 1964 Helsinki Declaration and were approved by the Ethics Committee of The Fifth Affiliated Hospital of Guangzhou Medical University (No. KY01-2020-03-05). Informed consent was obtained from all individual participants prior to any study-related procedures. All samples were immediately snap-frozen in liquid nitrogen and stored at −80 °C.

### Cell culture and transfection

Human HCC cell lines (Hep3B, SK-HEP-1, MHCC97L, MHCC97H, Huh7), normal human liver cells (LO2) and HEK293 cells were obtained from American Type Culture Collection (ATCC) (Manassas, VA, USA). All cells in this study were maintained in high glucose DMEM (Gibco, Carlsbad, CA, USA) supplemented with 10% FBS (Gibco, Carlsbad, CA, USA), 100 μg/mL penicillin and 100 U/mL streptomycin. The culture environment maintained at 37 °C containing 5% CO_2_.

For cell transfection, short hairpin RNA (shRNA) target circRNA_104348 or negative control (sh-NC), pcDNA3.1(+) CircRNA Vector for overexpressing circRNA_104348, miR-187-3p mimic, inhibitor, and their control plasmids (NC mimic, NC inhibitor) were purchased from VectorBuilder (Guangzhou, China). Cells were transfected with Lipofectamine 3000 (Invitrogen, Carlsbad, CA, USA).

### qRT-PCR and RNase R treatment

Total RNA in this study was isolated from cultured cells and tissues using Trizol reagent (Invitrogen, USA). Then, extracted RNAs were reverse-transcribed into cDNA by using Prime Script™ RT kit (Takara, Dalian, China). In addition, for miRNAs, MicroRNA Reverse Transcription Kit (Takara Biotechnology, Japan) were used to perform reverse transcription. The primers used in the current experiments were deigned and purchased from Geneseed Biomart (Guangzhou, China) (Table 1). qRT-PCR was carried out on ABI 7500 fast PCR System (Carlsbad, CA, USA) with a SYBR green PCR Master Mix (TOYOBO, Japan). GAPDH and U6 applied as internal references for mRNAs and miRNAs, respectively. The relative expressions were calculated with 2^–ΔΔCT^ method. For RNase R treatment, 1 unit of RNase R was added to digest 1 μg of RNA for 15 min at 37 °C.

**Table 1:** Primers used in the present study.

Gene	Primer sequence
miR-187-3p	forward: 5′-CACAGGACCCGGGCG-3′
	reverse: 5′-CCGGCTGCAACACAAGAC-3′
miR-665	forward: 5′-CTCGCTTCGGCAGCACA-3′
	reverse: 5′-CAGTGCGTGTCGTGGAGT-3′
U6	forward: 5′-CTCGCTTCGGCAGCACA-3′
	reverse: 5′-AACGCTTCACGAATTTGCG′
hsa_circRNA_104348	forward: 5′-TCTGTGTGTCAAAGCAAGGC-3′
	reverse: 5′-AGATGCCACTGAATCACCCA-3′
RTKN2	forward: 5′-CTCATGGTGTGCAATGCTCG-3′
	reverse:5′-ACTGCAGATAGAGAAATGGACTT-3′
GAPDH	forward: 5′-CTCTGCTCCTCCTGTTCGAC-3′reverse: 5′- GCGCCCAATACGACCAAATC-3′

### Fluorescence in situ hybridization (FISH)

For FISH assay, Fluor 488-labeled probe for detecting hsa_circRNA_104348 and Alexa Fluor 555-labeled probes for detecting miR-187-3p were synthesized by GenePharma. After fixation, cells were incubated with pre-hybridization buffer, then, hybridization was conducted at 55 °C for 2 h. Afterwards, the nuclei were stained with DAPI. The probe signals were determined with the FISH Kit (RiboBio, Guangzhou, China) based on the manufacturer’s instructions. Leica SP8 laser scanning confocal microscope was used for capturing images. The sequence of hsa_circRNA_104348 FISH was: 5′-CCTGTGAAAGTGTGCCCAGGGCTCCG-3′.

### CCK-8

Cell viability in different groups was tested by CCK-8 assay (Dojindo, Japan) based on the manufacturer’s protocol. A density of 3 × 10^3^ cells/well was seeded in 96‐well plates. Before testing, 10 μL of CCK-8 regent was added to the each well. After incubation, absorbance of cells was captured and recorded at 490 nm using a Varioskan Flash system (Thermo, USA) at different time points (24, 48, 72 h).

### Colony formation assay

Different groups of transfected HCC cells were seeded into six‐well plates. After 2 weeks, 10% formaldehyde was used for fixing cell colonies, then, fixed colonies were stained with 0.5% crystal violet. Camera (Olympus, Tokyo, Japan) was used for photographing colonies.

### Cell apoptosis assay

EdU staining was utilized to determine cell apoptosis. Briefly, HCC cells were treated with culture medium containing 20 μM EdU reagent. Then, cells were incubated in an incubator for 2 h, afterwards, they were fixed with paraformaldehyde. The nuclei were stained with DAPI. The percentage of EdU-positive cells were quantified and analyzed.

Flow cytometer assay was also used for detecting cell apoptosis. Briefly, cells were suspended in 600 μl flow cytometry binding buffer, then, at the room temperature, they were stained with 5 μl Annexin V/FITC and 5 μl propidium iodide (PI) in dark for 15 min. Results were analyzed using FlowJo 7.6 software.

### Wound-healing assay

Different groups of transfected HCC cells were seeded in a six-well plate and grew until ~80% confluence. The cell monolayer was wounded by scratching with a 200 µl pipette tip. The movement of cells was captured by an olympus microscope (Olympus, Tokyo, Japan) at 0 and 24 h, respectively.

### Transwell assay

Transwell chambers (Corning, New York, USA) were used to perform invasion assay. A total amount of 200 µL HCC cell-suspended fluid with a density of 2 × 10^4^ were added in upper chamber. Meanwhile, 600 µL culture medium with 20% FBS was added into the lower chamber. The invaded cells were fixed with 4% paraformaldehyde and stained with 0.5% crystal violet, calculated under an olympus microscope after 24 h.

### Immunofluorescence (IF)

IF staining was performed using transfected HCC cells on poly-lysine‐coated glass coverslips. Subsequently, 10% BSA (Amersco, USA) was used for blocking, then, primary antibody were added: anti-E-cadherin (Abcam, #ab194982, 1:1000), anti-N-cadherin (Abcam, #ab76057, 1:1000) at 4 °C overnight. Next, cells were incubated with a FITC-conjugated anti-rabbit secondary antibody (FITC, Invitrogen, CA), Alexa-conjugated anti-goat secondary antibody (Alexa647, Invitrogen, CA) for 1 h. Finally, cells were stained with diamidino-2-phenylindole (DAPI; 1:1000; Beyotime, China) for 15 min. Data were acquired and analyzed thereafter.

### Dual-luciferase reporter assay

HEK‐293T cell was cultured and seeded into 24-well plates. Then the pmirGLO reporter vector (Promega) carrying wild‐type (WT) or mutant type (MUT) has_circ_104348 and RTKN2 was transfected into HEK‐293T cell combination with miR‐187–3p mimics or NC mimics using Lipofectamine 2000 (Invitrogen, USA).

### RNA immunoprecipitation (RIP) assay

For RIP assay, Magna RIP kit (Millipore, Billerica, MA, USA) was used. Briefly, cell lysate was treated with RIP buffer containing magnetic beads conjugated with human anti-Ago2 antibody (Millipore, Billerica, MA, USA), or negative control IgG. Beads were washed with wash buffer, and the complexes were incubated with 0.1% SDS/proteinase K to remove proteins. qRT-PCR assay was carried out afterwards.

### Western blot assay

Different groups of HCC cells were lysed on ice with RIPA buffer. Then, protein lysates were electrophoresed on 10% SDS polyacrylamide gels, transferred onto PVDF membranes (Millipore, USA), and blocked with 5% skim milk. Afterwards, primary antibodies: anti-RTKN2 (Abcam, #ab251807, 1:1000), anti-β-catenin (Abcam, #ab16051, 1:1000), anti-PCNA (Abcam, #ab29, 1:1000), anti-CyclinD1 (Abcam, #ab134175, 1:1000), anti-c-Myc (Abcam, # ab32072, 1:1000), anti-Bcl-2 (Abcam, # ab185002, 1:1000), anti-Bax (Abcam, # ab32503, 1:1000), anti-MMP-7 (Abcam, # ab207299, 1:1000), anti-E-cadherin (Abcam, #ab1416, 1:500), anti-N-cadherin (Abcam, #ab76057, 1:1000) were added at 4 °C overnight. Subsequently, secondary antibodies (1:1,000; Millipore, USA) was incubated for 1 h. Data was obtained and calculated using ImageJ software.

### In vivo subcutaneous and orthotopic implantation model

Animal-related experiment was approved by the Ethics Committee of The Fifth Affiliated Hospital of Guangzhou Medical University (No. KY01-2020-03-05). For the subcutaneous implantation model, Huh7 cells with stable transfected sh-circRNA or sh-NC were inoculated subcutaneously in the right flank of the nude mice (male BALB/c, 4–5 weeks old). Tumor volume was calculated every 5 days and tumor weight was weighted at the end-point of the experiment. For orthotopic implantation, Huh7 cell transfected with sh-circRNA or sh-NC were luciferase labeled, then injected orthotopically into the left liver lobe of nude mice. 4 weeks later, tumor growth and lung metastases were detected with the IVIS 100 Imaging System (Xenogen). Moreover, lung tissue was subjected for hematoxylin and eosin (H&E) staining.

### Immunohistochemistry assay (IHC)

Paraffin sections of xenograft tumor tissues were cut with a thickness of 4 μm. Sections were incubated with primary antibodies: anti-RTKN2 (Abcam, #ab251807, 1:1000), anti-β-catenin (Abcam, #ab16051, 1:1000), anti-Ki67 (Abcam, #ab245113, 1:1000), anti-E-cadherin (Abcam, #ab1416, 1:500), and anti-N-cadherin (Abcam, #ab76057, 1:1000). The temperature was maintained at 4 °C overnight. Sections were co-incubated with HRP‐polymer‐conjugated secondary antibodies after washing with phosphate‐buffered saline, then, they were immunostained using DAB plus kit.

### Statistical analysis

Data were presented as the mean values ± the standard deviation (means ± SD). Data were statistically analyzed using SPSS 20.0 (IBM Corp., Armonk, NY, USA). Statistical significance was determined by one-way ANOVA test or Student’s *t*-test. Moreover, Pearson’s correlation coefficient and Kaplan–Meier analysis were used for analyzing statistical correlation and survival curves, respectively. *P* < 0.05 was presented as statistical significance.

## Results

### Has_circ_104348 was highly expressed in HCC tissue and cells

**Fig. 1:**
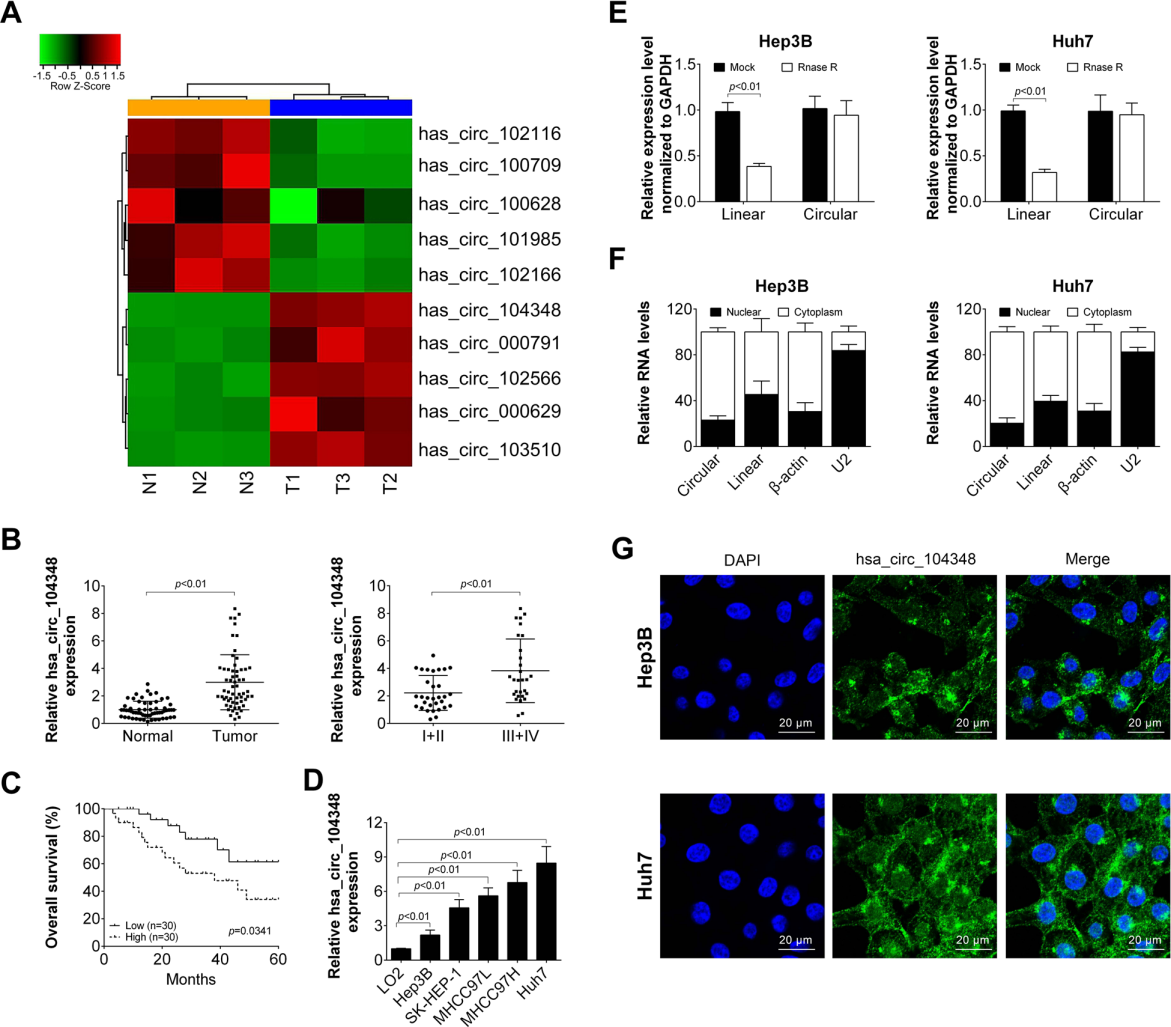
Has_circ_104348 was highly expressed in HCC tissue and cells. **A** Heatmap of upregulated circRNAs in HCC tissues. **B** The expression of has_circ_104348 was examined in HCC samples and normal tissues as well as in different clinical stages by qRT-PCR. **C** The 60 HCC samples were divided into high and low groups based on has_circ_104348 expression. Next, Kaplan–Meier survival analyses were conducted to determine the overall survival rate. **D** The expression of has_circ_104348 was detected in HCC cell lines (Hep3B, SK-HEP-1, MHCC97L, MHCC97H, Huh7) and normal liver cells (LO2) by qRT-PCR. **E** ELMO1 mRNA and circular has_circ_104348 were detected, RNA samples were treated with RNase R or mock treated without the enzyme. **F** The expression of has_circ_104348 was determined by qRT-PCR in cytoplasm or nuclear in HCC cells. **G** The localization of has_circ_104348 was detected by RNA FISH. The scale bars were 20 μm. Data were expressed as mean ± SD.

Firstly, three pairs of HCC tissues and controlled peritumor tissues were used to analyze the expression of circRNAs by qRT-PCR. Results of heat map showed that there were five circRNAs up-regulated in HCC tissues (has_circ_104348, has_circ_000719, has_circ_102566, has_circ_000629, has_circ_103510), meanwhile, five circRNAs were significantly downregulated in HCC tissues compared with control (Fig. 1A). In the current study, we selected has_circ_104348 as our protagonist. The expression levels of has_circ_104348 was significantly up-regulated in tumor tissue compared with that in control (Fig. 1B, *P* < 0.01, *n* = 60). Moreover, in advanced HCC stage (III + IV), levels of has_circ_104348 was markedly more expressed than that in early stage (Fig. 1B, *P* < 0.01). Next, we investigated the correlation between has_circ_104348 expression and prognosis of HCC patients. As shown in Fig. 1C, Kaplan–Meier survival analysis revealed that patients with higher level of has_circ_104348 expression had a lower survival rate (Fig. 1C). In addition, the association between has_circ_104348 expression and other clinical features of HCC patients was analyzed (Table 2). We found that tumor size, lymph node involvement as well as TNM stage were correlated with expression of has_circ_104348. To further explore the biologic roles of has_circ_104348, the expression of has_circ_104348 was also detected in HCC cells as well as in normal liver cells. Notably, in all HCC cells, including Hep3B, SK-HEP-1, MHCC97L, MHCC97H, Huh7 cells, the expression of has_circ_104348 was significantly up-regulated in HCC cells than in LO2 cells (Fig. 1D, *P* < 0.01). Based on this result, we selected Hep3B and Huh7 cells to perform further experiments in order to obtain full expression spectrum of has_circ_104348 in HCC cells. Next, the expression of linear ELMO1 mRNA and circular has_circ_104348 were detected by qRT-PCR in Hep3B and Huh7 cell lines, respectively. Results confirmed that circular has_circ_104348 was not affected by Rnase R. Furthermore, the localization of circular has_circ_104348 was detected by qRT-PC and RNA-FISH (Fig. 1F, G). Results demonstrated that has_circ_104348 was mainly expressed in cytoplasm in HCC cells rather than in nuclear. Taken together, our data revealed that has_circ_104348 was highly expressed in HCC tissue and cells, correlated with poor prognosis of HCC patients, localized mainly in cytoplasm of HCC cells.

**Table 2:** Association between clinical features and hsa_circ_104348 expression of hepatocellular carcinoma patients.

Clinical features	hsa_circ_104348 expression		*P* value
	Low expression (<median)	High expression (≥median)	
Number	30	30	
*Sex*			0.796
Male	16	15	
Female	14	15	
*Age (years)*			0.774
<55	22	21	
≥55	8	9	
*Tumor size* (*cm*)			0.035*
<5	16	8	
≥5	14	22	
*Lymph node involvement*			0.009*
Absent	18	8	
Present	12	22	
*HBV infection*			0.121
Yes	19	13	
No	11	17	
*TNM stage*			0.020*
I+II	20	11	
III+IV	10	19	

### Has_circ_104348 promoted proliferation, suppressed apoptosis of HCC cells

**Fig. 2:**
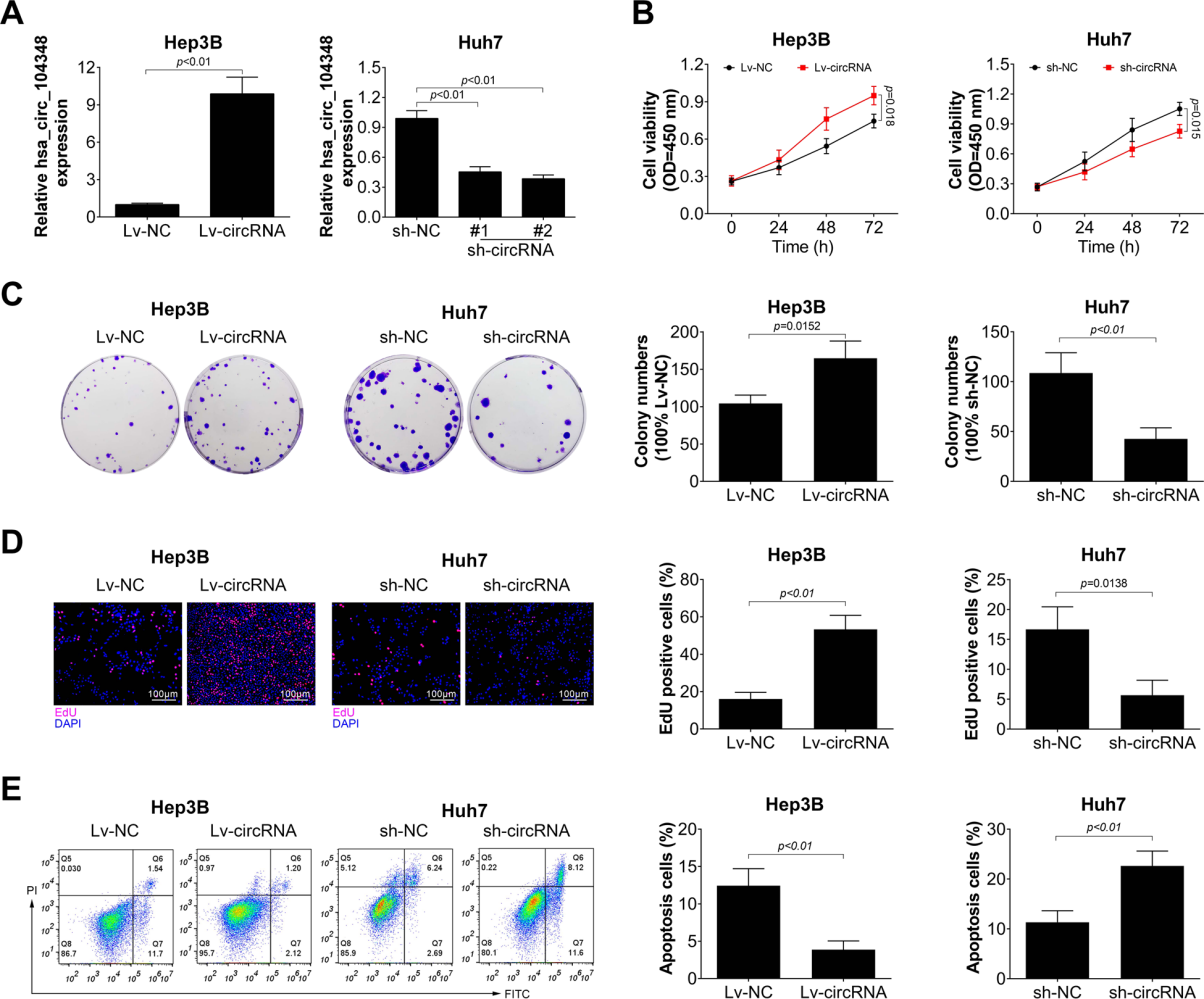
Has_circ_104348 promoted proliferation, suppressed apoptosis of HCC cells. **A** The expression of has_circ_104348 was determined by qRT-PCR in has_circ_104348 overexpression or knockdown HCC cells. **B**–**D** The effect of has_circ_104348 on HCC cell proliferation was determined by CCK-8 (**B**), colony formation (**C**), and EdU assay (**D**). The apoptosis of HCC cells was measured by staining with Annexin V/PI, followed by FACS analysis. The scale bars were 100 μm. Data were expressed as mean ± SD.

To further investigate the pathologic role of has_circ_104348, we first established the has_circ_104348 overexpression and knockdown systems by using Lv-circRNA or sh-circRNA in Hep3B and Huh7 cell lines, respectively. As shown in Fig. 2A, the systems were successfully established (*P* < 0.01). Then, cell viability was determined in different groups by CCK-8. Results demonstrated that overexpression of has_circ_104348 increased HCC cell viability, however, knockdown of has_circ_104348 markedly inhibited cell viability (Fig. 2B, *P* < 0.05). Subsequently, colony formation assay was conducted to identify the proliferation of HCC cells in either Lv-circRNA or sh-circRNA group. As shown in Fig. 2C, the colony numbers were much more in has_circ_104348 overexpression group than in control (*P* < 0.05). On the contrary, in has_circ_104348 knockdown group, the colony numbers were much less (*P* < 0.01). Moreover, the results of EDU staining also demonstrated that overexpression of has_circ_104348 could promote proliferation of HCC cell (Fig. 2D, *P* < 0.01), sh-circRNA showed the opposite result (Fig. 2D, *P* < 0.05). In addition, cell apoptosis was determined by flow cytometry. We found that apoptosis rate of HCC cells was inhibited by overexpression of has_circ_104348, while it was promoted by knockdown of has_circ_104348 (Fig. 2E, *P* < 0.01). Overall, these results demonstrated that has_circ_104348 promoted proliferation, but inhibited apoptosis of HCC cells.

### Has_circ_104348 promoted migration and invasion of HCC cells

**Fig. 3:**
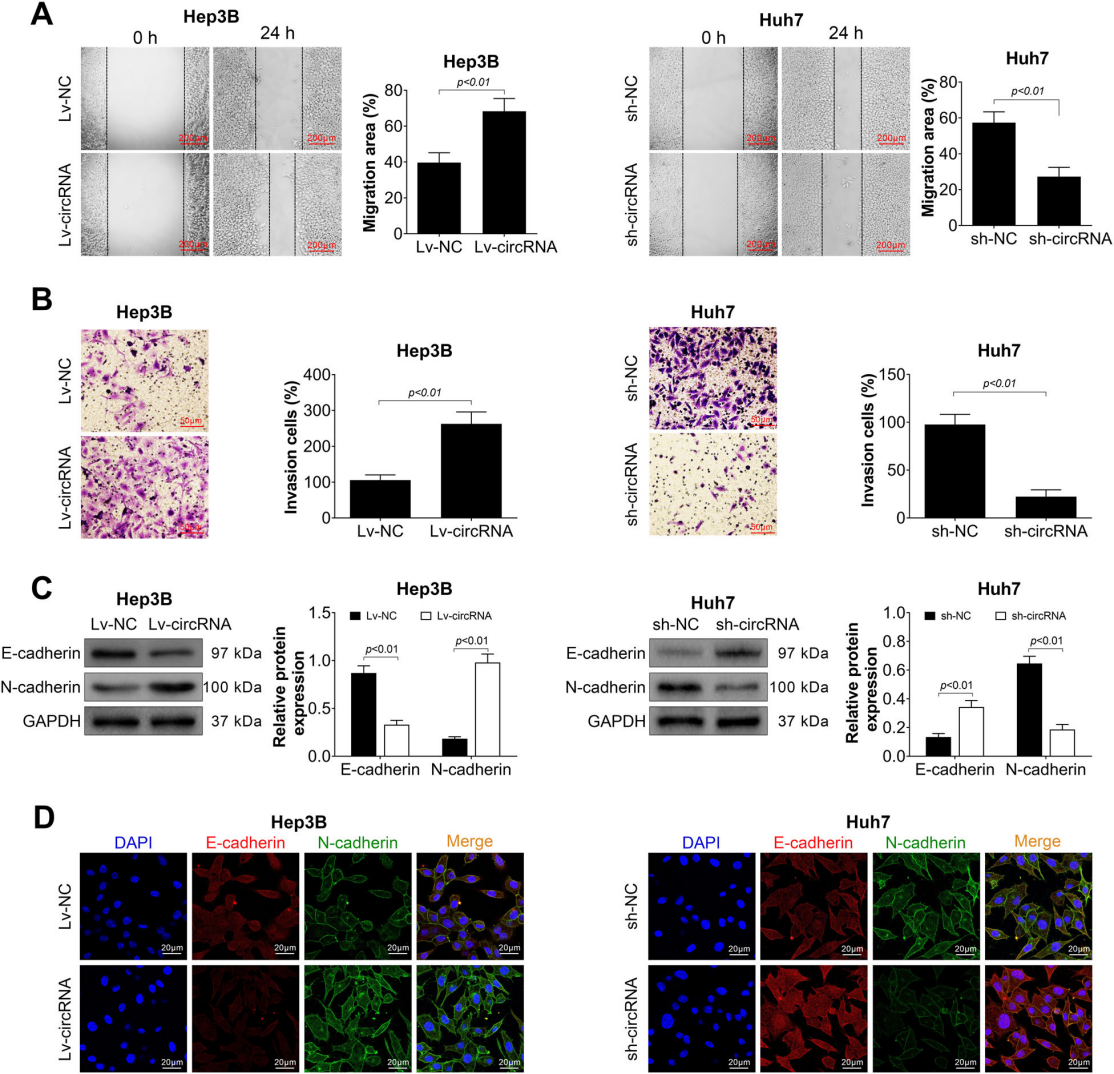
Effect of has_circ_104348 on HCC cell migration and invasion. **A** and **B** The effect of has_circ_104348 on HCC cell migration and invasion was determined by wound healing assay (**A**) and transwell assay (**B**). The scale bars were 200 μm. **C** and **D** The expression of E-cadherin and N-cadherin was tested by western blot (**C**) and IF (**D**). The scale bars were 20 μm. Data were expressed as mean ± SD.

Subsequently, has_circ_104348 on migration and invasion of HCC cells was determined by wound healing assay and Transwell assay. As shown in Fig. 3A, B, overexpression of has_circ_104348 could significantly promote the migration rate of HCC cells (*P* < 0.01). However, with sh-circRNA, the migration ability of HCC cells was inhibited (*P* < 0.01). For cell invasion, tranwell assay showed the similar tendency (Fig. 3B, *P* < 0.01). In addition, bio-makers of cell invasion include E-cadherin and N-cadherin were determined in has_circ_104348 overexpression and knockdown system by using Western blot and immune fluorescence assay, respectively. Results showed that overexpression of has_circ_104348 markedly suppressed the expression of E-cadherin, increased the expression of N-cadherin (Fig. 3C and D, *P* < 0.01). On the contrary, in has_circ_104348 knockdown system, the expression of N-cadherin was suppressed while expression of E-cadherin was promoted (Fig. 3C and D, *P* < 0.01). To conclude, these data confirmed that has_circ_104348 could promote migration and invasion of HCC cells.

### The expression of has_circ_104348 was negatively correlated with the expression of miR-187-3p

**Fig. 4:**
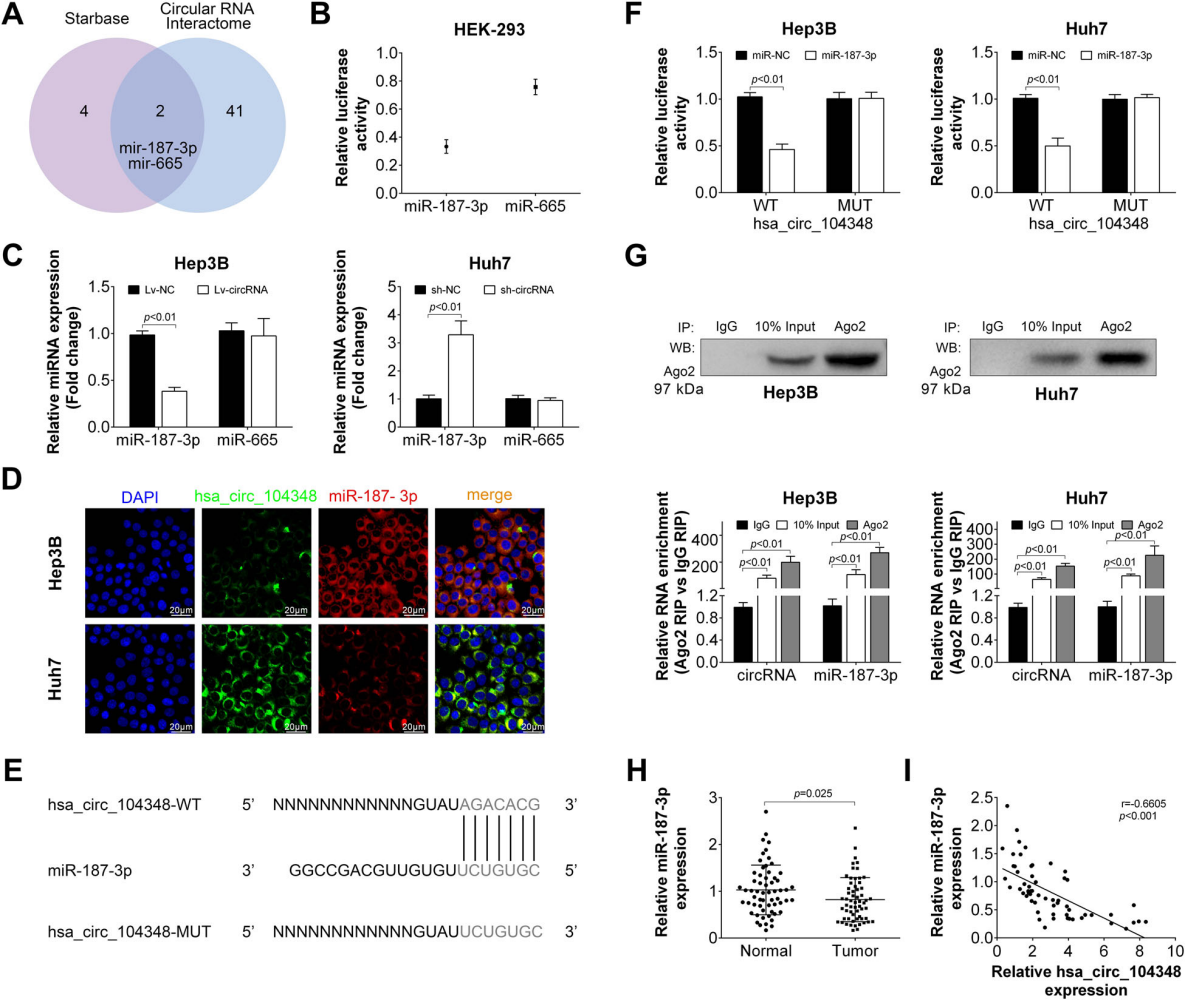
The expression of has_circ_104348 was negatively correlated with the expression of miR-187- 3p. **A** Overlap region showed the predicted miRNAs from Starbas and Circular RNA Interactome. **B** Relative luciferase activity of miR-187-3p and miR-665 was detected by dual-luciferase reporter assay. **C** The expression of miR-187-3p and miR-665 was determined in has_circ_104348 overexpression or knockdown system. **D** The location of has_circ_104348 and miR-187-3p were detected by FISH. The scale bars were 20 μm. **E** The potential binding site of has_circ_104348 and miR-187-3p. **F** Dual-luciferase reporter assay was performed to confirm the binding site of has_circ_104348 and miR-187-3p. **G** RIP assay was used to test the combination of has_circ_104348 and miR-187-3p. **H** The expression of miR-187-3p was determined in tumor or paired normal tissues by qRT-PCR. **I** The correlation analysis between has_circ_104348 and miR-187-3p. Data were expressed as mean ± SD.

To better understand the molecular mechanism of has_circ_104348, we used bioinformatics tools to predict the possible miRNA target of has_circ_104348. We used Circular RNA Interactome (https://circinteractome.nia.nih.gov/) and Starbas (http://starbase.sysu.edu.cn/) to predict the target of has_circ_104348. As shown in Fig. 4A, two miRNAs, miR-187-3p and mir-665 were the potential target of has_circ_104348. Next, the relative luciferase activity of miR-187-3p and miR-665 was detected by dual-luciferase reporter assay. Result showed that the relative luciferase activity of miR-665 was higher than miR-187-3p (Fig. 4B). Then, the relative expression of these two miRNAs were determined in has_circ_104348 overexpression and knockdown system. As shown in Fig. 4C, after transfected with Lv-circRNA or sh-circRNA, the level of miR-187-3p was negatively affected (*P* < 0.01), while there was no change in expression of miR-665. Then, the location of has_circ_104348 and miR-187-3p were detected by FISH. Results indicated that both has_circ_104348 and miR-187-3p were most presented in cytoplasm (Fig. 4D). Based on these data, we hypothesized that miR-187-3p could be a target of has_circ_104348. We further found there was a potential binding site between has_circ_104348 and miR-187-3p (Fig. 4E). The hypothesis was confirmed by dual-luciferase reporter assay and RIP assay (Fig. 4F, G). Plasmids containing the potential miR-187-3p-binding sites: circ_101141 wild-type (WT) or mutated (MUT) was co-transfected with miR-NC or miR-187-3p mimic. Results revealed that in both Hep3B and Huh7 cell lines, the relative luciferase activity of has_circ_104348 WT with miR-187-3p mimic group was significantly lower than other group (Fig. 4F, *P* < 0.01), suggesting that miR-187-3p could be a direct target of has_circ_104348. In RIP assay, compared with control IgG, circRNA was preferentially enriched in miRNA ribonucleoprotein complexes containing Ago2 (Fig. 4G, *P* < 0.01). Additionally, the expression of miR-187-3p was determined in HCC tissues, results from qRT-PCR revealed that miR-187-3p was significantly decreased in HCC tissues (Fig. 4H, *P* = 0.025), and expression level of miR-187-3p was negatively correlated with expression level of has_circ_104348 (Fig. 4I, *r* = −0.6605, *P* < 0.001). To sum up, our data indicated that has_circ_104348 was negatively correlated with the expression of miR-187-3p.

### RTKN2 was a direct target of miR-187- 3p

**Fig. 5:**
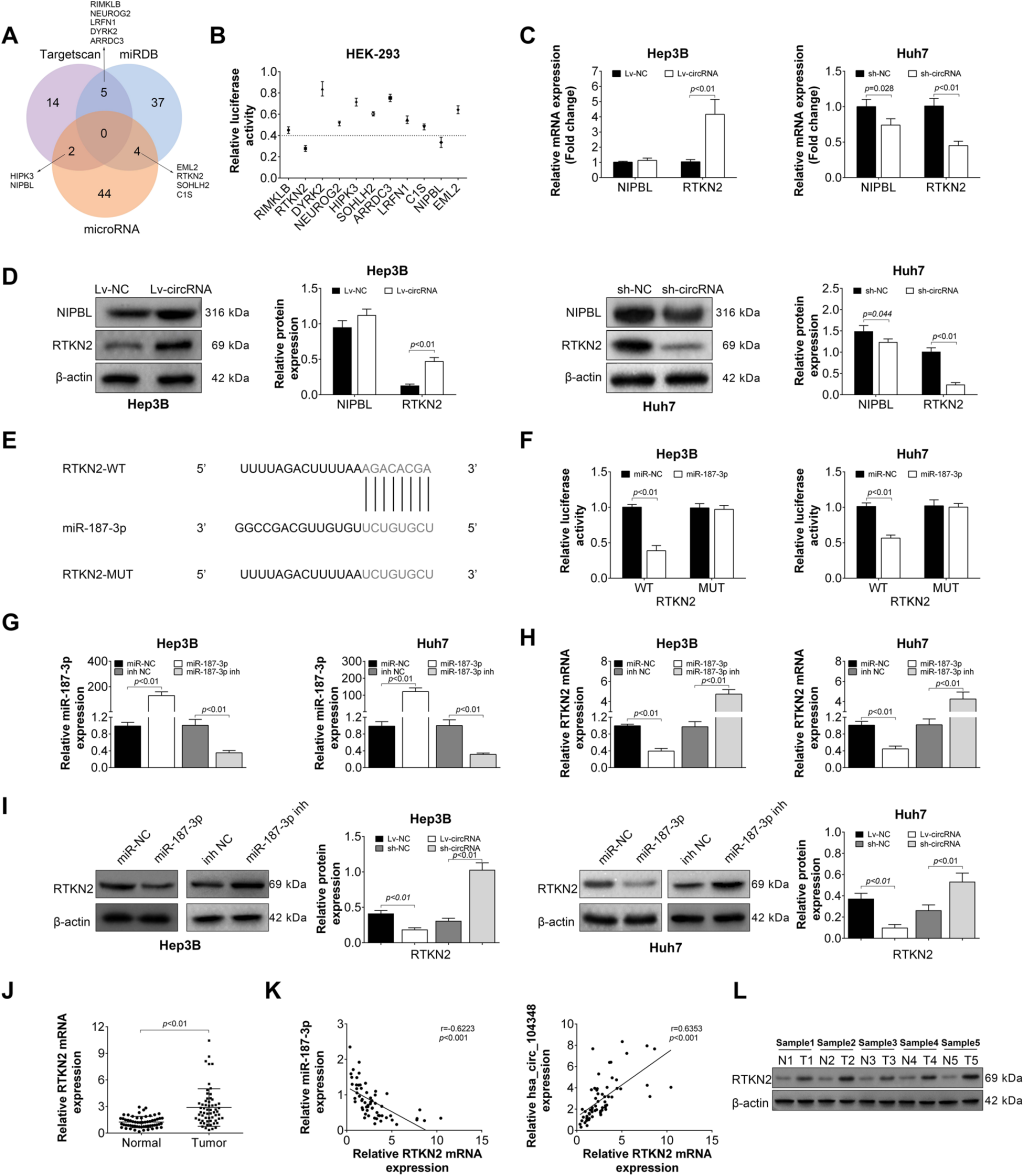
RTKN2 was a direct target of miR-187-3p. **A** The potential target of miR-187-3p was predicted by Targetscan, miRDB, and microRNA. **B** Relative luciferase activity of 11 genes was detected by dual-luciferase reporter assay. **C** and **D** The mRNA and protein level of RTKN2 and NIPBL in has_circ_104348 overexpression or knockdown systems was determined by qRT-PCR (**C**) or western blot (**D**). **E** RTKN2 3′-UTR wide-type and the mutated-type in the miR-187-3p-binding sites were shown. **F** Luciferase activity of HEK293 cells co-transfected with miR-187-3p mimics or NC mimics and luciferase reporters containing RTKN2 3′-UTR WT or RTKN2 3′-UTR MUT transcript were determined by dual-luciferase reporter assays. **G** The transfection efficiency of miR-187-3p mimics and inhibitors was tested. **H** and **I** The levels of RTKN2 transfected with miR-187-3p mimics or NC mimics, as well as miR-187-3p inhibitor or NC inhibitor in HCC cells were analyzed by qRT-PCR (**H**) and western blot (**I**). **J** The expression of RTKN2 and corresponding normal tissues were detected by qRT-PCR. **K** The correlation between RTKN2 and miR-187-3p, as well as RTKN2 and has_circ_104348 expression was analyzed. **L** he protein level of RTKN2 in HCC patients and normal subjects were measured in five samples by western blot. Data were expressed as mean ± SD.

Then, a series of experiments were performed to better understand the underlying mechanisms of miR-187-3p and its down-stream factors. Firstly, bioinformatics tools (Targetscan, miRDB, and microRNA) were used to predict the potential targets of miR-187-3p. As shown in Fig. 5A, a total number of 11 genes were selected for further dual-luciferase reporter assay experiments. The results demonstrated that among those genes, the relative luciferase activity of RTKN2 and NIPBL were significantly decreased, suggesting that the expression of RTKN2 and NIPBL may be mostly affected by miR-187-3p (Fig. 5B). Then, the expression of RTKN2 and NIPBL mRNA was detected in has_circ_104348 overexpression and knockdown system, respectively. We found that the expression of RTKN2 mRNA was remarkably up-regulated in Lv-circRNA group, while down-regulated in sh-circRNA group (Fig. 5C, *P* < 0.01). However, the expression of NIPBL was only affected by knockdown of has_circ_104348 (Fig. 5C, *P* < 0.01), there was no change in Lv-circRNA group (Fig. 5C). The protein expression of RTKN2 and NIPBL showed the similar tendency as their mRNA (Fig. 5D, *P* < 0.01). Additionally, the relationship between miR-187-3p and RTKN2 was explored. Figure 5e shows the potential binging site of these two factors. Results from dual-luciferase reporter assay confirmed that RTKN2 could be a target of miR-187-3p (Fig. 5F). Then, miR-187-3p overexpression and knockdown systems were successfully established in Hep3B and Huh7 cell lines, respectively (Fig. 5G, *P* < 0.01). The expression of RTKN2 mRNA or protein was determined in miR-187-3p overexpression and knockdown systems, respectively. Results showed that expression of RTKN2 mRNA or protein was negatively regulated by miR-187-3p (Fig. 5H, I). In human tissues, the expression of RTKN2 mRNA was also determined. The expression of RTKN2 was significantly up-regulated in tumor tissues compared with control (Fig. 5J, *P* < 0.01). Correlation analysis revealed that the expression of RTKN2 was positively correlated with miR-187-3p, while negatively correlated with has_circ_104348 (Fig. 5K). In addition, expression of protein RTKN2 was detected in five pairs of tumor tissues or normal tissues. As shown in Fig. 5l, the protein RTKN2 was mainly expressed in tumor tissues. To sum up, results above suggested that RTKN2 could be a target of miR-187-3p, and expression of RTKN2 can be also affected by has_circ_104348.

### Hsa_circ_104348 regulated miR-187-3p to affect proliferation, migration, invasion, and apoptosis of HCC cells, and may have effect on Wnt/β-catenin signaling pathway

**Fig. 6:**
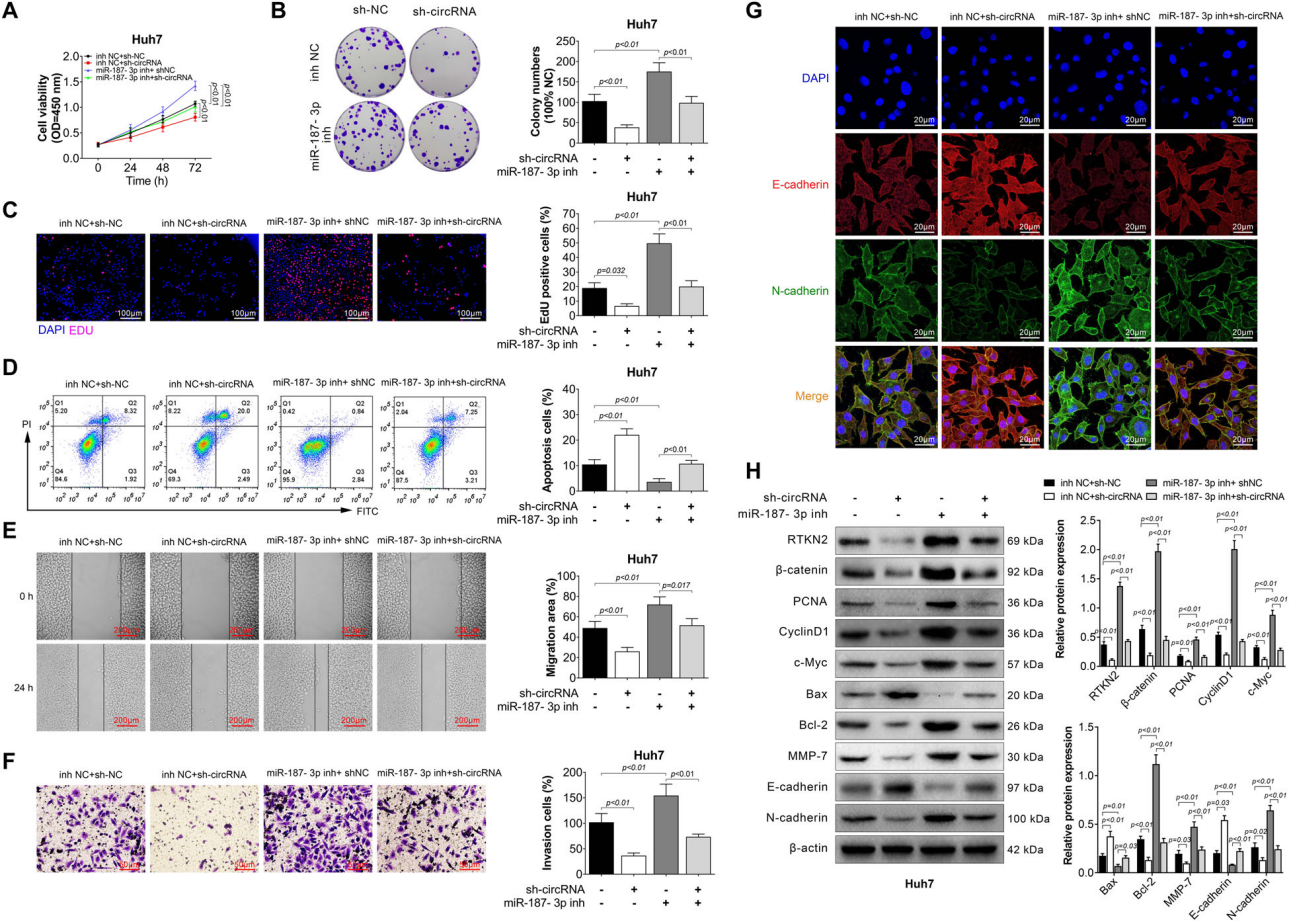
Hsa_circ_104348 regulated miR-187-3p to affect proliferation, migration, invasion, and apoptosis of HCC cells, and may have effect on Wnt/β-catenin signaling pathway. Huh7 cells were co-transfected with sh-circRNA or sh-NC and miR-187-3p inhibitor or inhibitor NC: **A** and **B** Cell proliferation abilities were determined by CCK-8 assay (**A**) and colony formation assay (**B**). **C** and **D** Cell apoptosis was determined by EdU staining (**C**) and flow cytometry assay (**D**). The scale bars were 100 μm. **E** and **F** Cell migration and invasion abilities were checked by wound healing assay (**E**) and transwell assay (**F**). The scale bars were 50 μm. **G** The expression of E-cadherin, N-cadherin was determined by IF. The scale bars were 20 μm. **H** The expression of RTKN2, β-catenin, PCNA, CyclinD1, c-Myc, Bcl-2, Bax, MMP-7, E-cadherin, N-cadherin was determined by western blot. Data were expressed as mean ± SD.

The further step was to clarify the effect of hsa_circ_104348 and miR-187-3p on phenotype of HCC cells. Sh-circRNA or sh-NC and miR-187-3p inhibitor or inhibitor-NC were co-transfected in Huh7 cells. Results of CCK-8 assay revealed that cell viability was greatly suppressed by knockdown of hsa_circ_104348, while miR-187-3p could compromise this effect (Fig. 6A). Similarly, colony numbers were decreased by transfecting sh-circRNA, however, this affect can be reversed by miR-187-3p (Fig. 6B, *P* < 0.01). Then, apoptosis was detected in different groups by EdU staining and flow cytometry assay. As shown in Fig. 6C, D, knockdown of has_circ_104348 significantly promoted cell apoptosis, after transfecting of miR-187-3p, the apoptosis rate was decreased (*P* < 0.01). In would healing assay and transwell assay, the results demonstrated that knockdown of has_circ_104348 could inhibit cell migration and invasion abilities, however, this inhibitory effect was attenuated by down-regulation of miR-187-3p in Huh7 cells (Fig. 6E, F, *P* < 0.01). Moreover, bio-markers of cell migration, invasion, apoptosis as well as key factors in Wnt/β-catenin signaling pathway were determined by IF assay and western blot. We found expression of RTKN2, β-catenin, PCNA, CyclinD1, c-Myc, Bcl-2, MMP-7, N-cadherin was markedly down-regulated in circRNA knockdown group, while after transfecting miR-187-3p, the expression of these proteins was up-regulated (Fig. 6G, H, *P* < 0.01). However, the expression of Bax and E-cadherin have opposite tendency. Further, we also determined the expression of p-GSK-3β and LEF1, p-GSK-3β was up-regulated in circRNA knockdown group, its expression was significantly decreased after transfecting with miR-187-3p. However, the expression of LEF1 have opposite tendency (Fig. S1, *P* < 0.01). Overall, these data indicated that hsa_circ_104348 regulated miR-187-3p to affect proliferation, migration, invasion, and apoptosis of HCC cells, and may have effect on Wnt/β-catenin signaling pathway.

### Knockdown of hsa_circ_104348 inhibited HCC progression

**Fig. 7:**
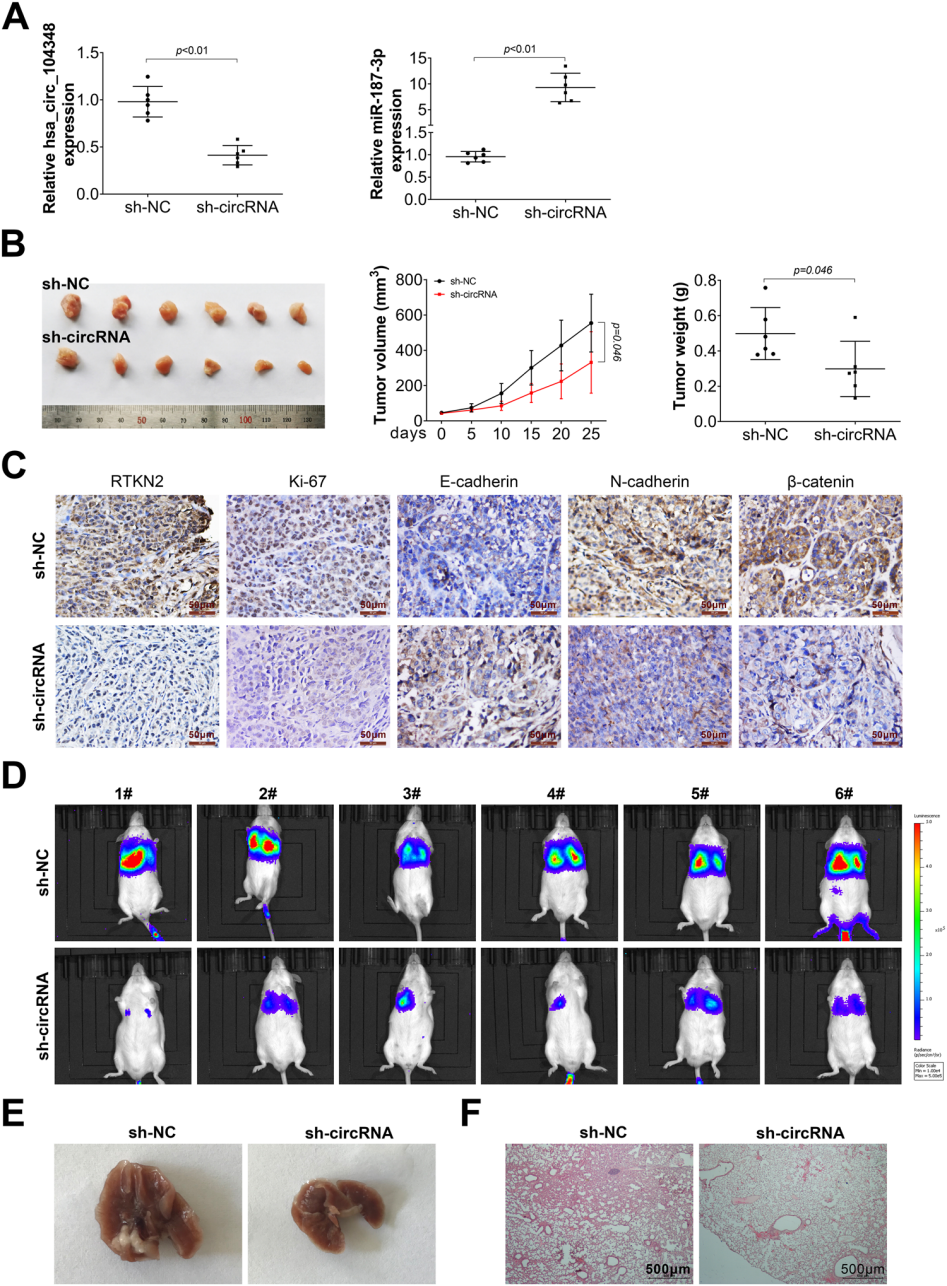
Knockout of hsa_circ_104348 inhibited HCC progression in mice treated with Huh7 cell transfected with sh-circRNA or sh-NC plasmid. **A** The expression of hsa_circ_104348 and miR-187-3p was detected by qRT-PCR. **B** The effect of hsa_circ_104348 silencing on tumor volume curve and tumor weight were analyzed. **C** The scale bars were 50 μm. The expression of RTKN2, β-catenin, Ki67, E-cadherin, N-cadherin was determined by IHC. **D** Lung metastasis was detected by in vivo images. **E** Metastatic focis from mice were shown. **F** HE-staining of pulmonary nodules from mice. The scale bars were 500 μm. Data were expressed as mean ± SD.

Finally, orthotopic xenograft mouse models were utilized to determine the effect of hsa_circ_104348 on tumorigenesis in animal model. Huh7 cells transfected with sh-circRNA or sh-NC plasmid were subcutaneously inoculated into male nude mice. The expression of hsa_circ_104348 and miR-187-3p was determined using qRT-PCR. It has shown that knockdown of hsa_circ_104348 could increase the expression of miR-187-3p in animal tissue (Fig. 7A). Then, tumor growth and volume were measured. Results showed that in sh-circRNA group, tumor growth and volume were slower and smaller than that in sh-NC group (Fig. 7B). IHC assay was conducted to detect the expression of RTKN2, Ki-67, E-cadherin, N-cadherin, and β-catenin. As shown in Fig. 7C, RTKN2, Ki-67, and N-cadherin were less expressed in hsa_circ_104348 knockdown group, while expression of E-cadherin has opposite result (Fig. 7C). Additionally, the lung metastasis was detected by in vivo images (Fig. 7D). Furthermore, metastatic focis and its HE-staining was shown in Fig. 7E, F. Data indicated that knockdown of hsa_circ_104348 inhibited tumorigenesis and lung metastasis.

## Discussion

With the development of high-throughput sequencing technology, more and more circRNAs have been discovered^23^. As a novel gene regulator, circRNAs exert their roles at the transcriptional or post-transcriptional level to regulate down-stream factors^24^. Increasing evidence have suggested that circRNAs could be differently expressed in tumor tissue and participate in HCC progression^25–27^. However, their functions remain not fully understood. In our study, by detecting expression of circRNAs in three pairs of HCC tissue, we have discovered hsa_circ_104348 was significantly up-regulated. Moreover, level of hsa_circ_104348 was closely related to the prognosis of patients with HCC, suggesting that hsa_circ_104348 might play an important role in HCC progression. Subsequently, functional experiments of hsa_circ_104348 during HCC progression were performed.

Gain-of-function experiments were firstly conduced in HCC cells, includes Hep3B and Huh7, to investigate the functional role of hsa_circ_104348. Results demonstrated that overexpressed hsa_circ_104348 could promote proliferation, migration, and invasion of HCC cells, while inhibit apoptosis of these cells. Data from gain-of-function experiments indicated that hsa_circ_104348 indeed plays a functional regulatory role in the pathogenesis of HCC.

The next step was to clarify the molecular mechanism of how hsa_circ_104348 exerts its functional role in HCC progression. To this end, we used the bioinformatics tools to predict the potential target of hsa_circ_104348, and finally confirmed miR-187-3p was one of the bio-target of hsa_circ_104348. As mentioned previously, miRNAs are also considered to be a class of non-coding RNA with a length of 22–25 nucleotides^28^. They exert their roles by targeting 3′-UTRs of mRNA post-transcriptionally to repress gene expression^29^. Among them, in published data, miR-187-3p was shown to be involved in various types of cancers. For instance, Yang et al. ^30^ used bioinformatics analysis to screen new biomarkers of colorectal cancer. They found miR-187-3p was one of the biomarkers that can predict the survival prognosis for patients with colorectal cancer. In non-small cell lung cancer (NSCLC), miR-187-3p was dramatically down-regulated, moreover, NSCLC cell growth was significantly suppressed by miR-187-3p^31^. In HCC, miR-187-3p was also confirmed to inhibit the metastasis and epithelial–mesenchymal transition (EMT) of HCC cells by regulating S100A4^32^. From these studies, we conclude that miR-187-3p may serve an inhibitory role to suppress progression of cancers. Not surprisingly, in our study, miR-187-3p was dramatically down-regulated in human HCC tissue. Moreover, expression of miR-187-3p can be regulated by hsa_circ_104348. In circRNA and miR-187-3p inhibitor co-transfected system, interference of miR-187-3p inhibitor could attenuate the effect of down-regulated hsa_circ_104348 on cell proliferation, migration, invasion as well as apoptosis, suggesting that hsa_circ_104348 could regulate miR-187-3p to affect the HCC progression. Moreover, key factors involved in Wnt/β-catenin pathway, e.g. β-catenin could also be affected by hsa_circ_104348 and miR-187-3p.

Rhotekin was known as a member of certain proteins containing a Rho-binding domain^33^. As a Rho effector, Rhotekin 2 (RTKN2) is highly expressed in different organs and tissues, such as spleen, thymus, lung, colon, and bone marrow^34,35^. Previous studies also have shown that RTKN2 was up-regulated in other types of cancer tissues and cells, e.g. ovarian cancer^36^, bladder cancer^37^. In HCC-related studies, Wei et al. reported that RTKN2 was overexpressed in HCC tissue. Knockdown of RTKN2 inhibited the levels of cell cycle-associated proteins, suppressed cell invasion, and induced cell apoptosis^38^. In the current study, by performing bioinformatic analysis, dual-luciferase reporter assay, qRT-PCR, we found that RTKN2 was a direct target of miR-187-3p. Results from western blot (Fig. 6H) revealed that expression of RTKN2 can be regulated by not only hsa_circ_104348, but also miR-187-3p.

The novel concept of competing endogenous RNAs (ceRNAs) was widely accepted in many circRNAs-related studies^39^. In these studies, circRNAs might act as “sponge” in the regulatory network referring to circRNAs, miRNA, and their target genes^39–41^. In our study, hsa_circ_104348 serve as a ceRNA for miR-187-3p to further regulate the expression of RTKN2 as well as Wnt/β-catenin pathway. Further in in vivo study, we have confirmed that knockdown of hsa_circ_104348 inhibited tumorigenesis and lung metastasis.

In summary, our study provides the evidence of hsa_circ_104348 was highly expressed in HCC tissues and correlated with outcomes of patients with HCC. Moreover, it can act as a regulator in cell proliferation, migration, invasion, and apoptosis. Mechanistically, hsa_circ_104348 function as a ceRNA for miR-187-3p to modulate RTKN2 expression, as well as activating Wnt/β-catenin pathway. Hsa_circ_104348 might act as a potential biomarker for HCC prognosis.

## Supplementary information

Fig S1

Supplementary Figure Legends

cell identification reports of Hep3B

cell identification reports of HUH-7

## Data Availability

All data generated or analyzed during this study are included in this published article.
