# Supplementary figures and images for: CircRNA hsa_circRNA_104348 promotes hepatocellular carcinoma progression through modulating miR-187-3p/RTKN2 axis and activating Wnt/β-catenin pathway
Source: Cell Death Dis. 2020 Dec 14;11(12):1065. doi: 10.1038/s41419-020-03276-1 (PMC7734058; doi:10.1038/s41419-020-03276-1)

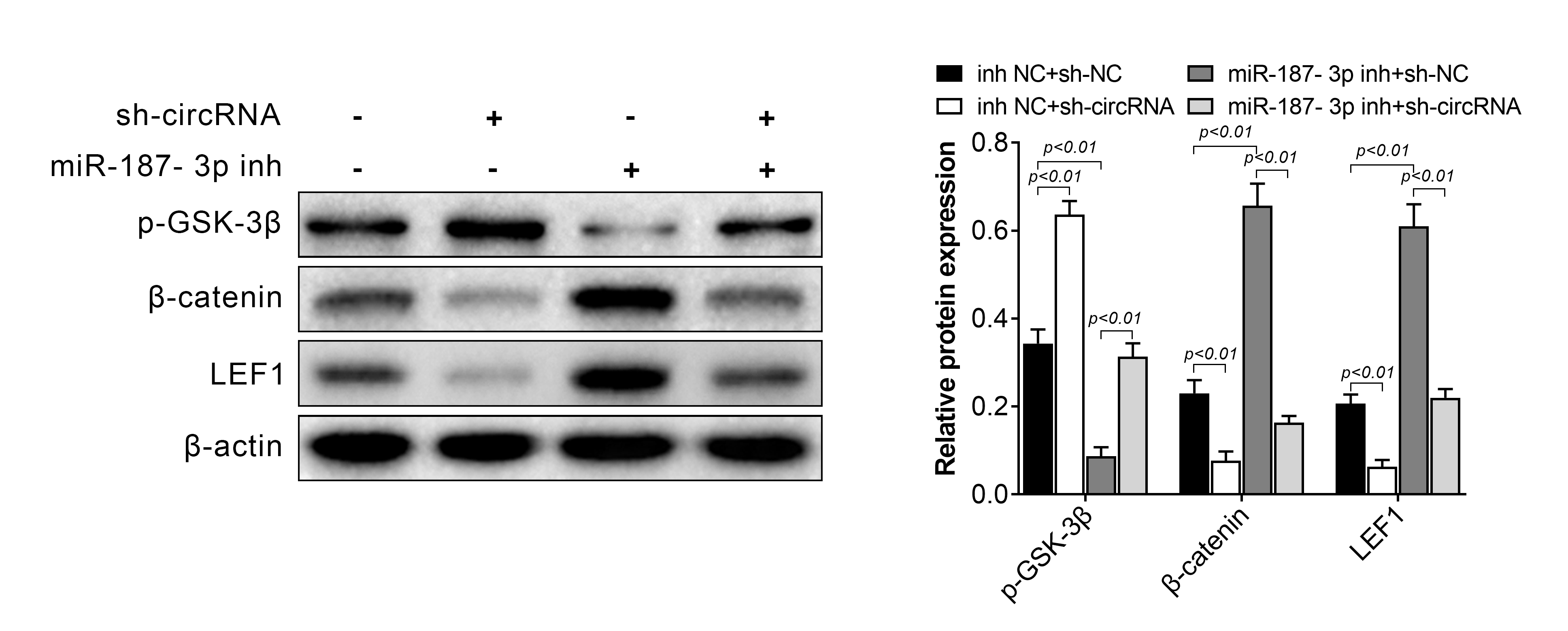

Supplement: Supplementary file 1 — Fig S1 [file 41419_2020_3276_MOESM1_ESM.jpg]
