# Supplementary material for: CircRNA hsa_circRNA_104348 promotes hepatocellular carcinoma progression through modulating miR-187-3p/RTKN2 axis and activating Wnt/β-catenin pathway
Source: Cell Death Dis. 2020 Dec 14;11(12):1065. doi: 10.1038/s41419-020-03276-1 (PMC7734058; doi:10.1038/s41419-020-03276-1)
Supplement: Supplementary file 2 — Supplementary Figure Legends [file 41419_2020_3276_MOESM2_ESM.docx]

**Figure S1 The expression of p-GSK-3β, β-catenin and LEF1 was determined by western blot.**
