# Supplementary material for: CircRNA hsa_circRNA_104348 promotes hepatocellular carcinoma progression through modulating miR-187-3p/RTKN2 axis and activating Wnt/β-catenin pathway
Source: Cell Death Dis. 2020 Dec 14;11(12):1065. doi: 10.1038/s41419-020-03276-1 (PMC7734058; doi:10.1038/s41419-020-03276-1)

细胞遗传质量鉴定检验报告

检品名称：细胞系

检验类型：STR 基因型检验

样品编号：

表 1 样本编号

客户样本编号	公司编号
211	20190315-37

样品数量： 1

样品性状： 细胞系

检测项目： STR

检测方法： 用 Axygen 的基因组抽提试剂盒提取 DNA，采用 20- STR 扩增方案扩增，在 ABI 3730XL 型遗传分析仪上对 STR 位点和性别基因 Amelogenin 进行检测。

检验结果：

(一) 检验基本情况

表 2：样本基因型检验结果

9		多等位基因	匹配细胞系	细胞库	EV 值	匹配说明
	20190315-37	无	hep3b2.1-7(hep3b)	ATCC	1	完全匹配

- 多等位基因指三等位及以上基因现象。
- 本次检测各细胞分型结果良好。

(二)各样本描述

- 20190315-37：该株细胞 DNA 分型在细胞系检索中找到<u>完全匹配</u>的细胞系，ATCC数据库显示细胞名为 **<u>HEP3B2.1-7(HEP3B)</u>**，细胞号对应 **<u>HB-8064</u>**，本次检测在该细胞系中<u>没有发现多等位基因</u>。

(三)样本分型结果

表 3：细胞 20190315-37 的 STR 位点和 Amelogenin 位点的基因分型结果

Marker	样本				细胞库信息		
	Allele1	Allele2	Allele3	Allele4	Allele1	Allele2	Allele3
D5S818	13	13			13	13	
D13S317	12	14			12	14	
D7S820	8	10			8	10	
D16S539	10	10			10	10	
VWA	17	17			17	17	
TH01	6	7			6	7	
AMEL	X	X			X	X	
TPOX	9	9			9	9	
CSF1PO	8	8			8	8	
D12S391	17	17					
FGA	18	18					
D2S1338	21	25					
D21S11	30	31					
D18S51	20	20					
D8S1179	12	12					
D3S1358	15	15					
D6S1043	12	17					
PENTAE	5	16					
D19S433	12.2	14					
PENTAD	12	14					

其他说明:

(一) 分型方案及位点分布:

附表：实验方案及位点

	方案 1	方案 2	方案 3	方案 4
1	TH01	TPOX	D3S1358	AMEL
2	D12S391	VWA	D13S317	D5S818
3	D7S820	D8S1179	D6S1043	D2S1338
4	CSF1PO	PENTAD	D16S539	D21S11
5	FGA		D19S433	D18S51
6	PENTAE			

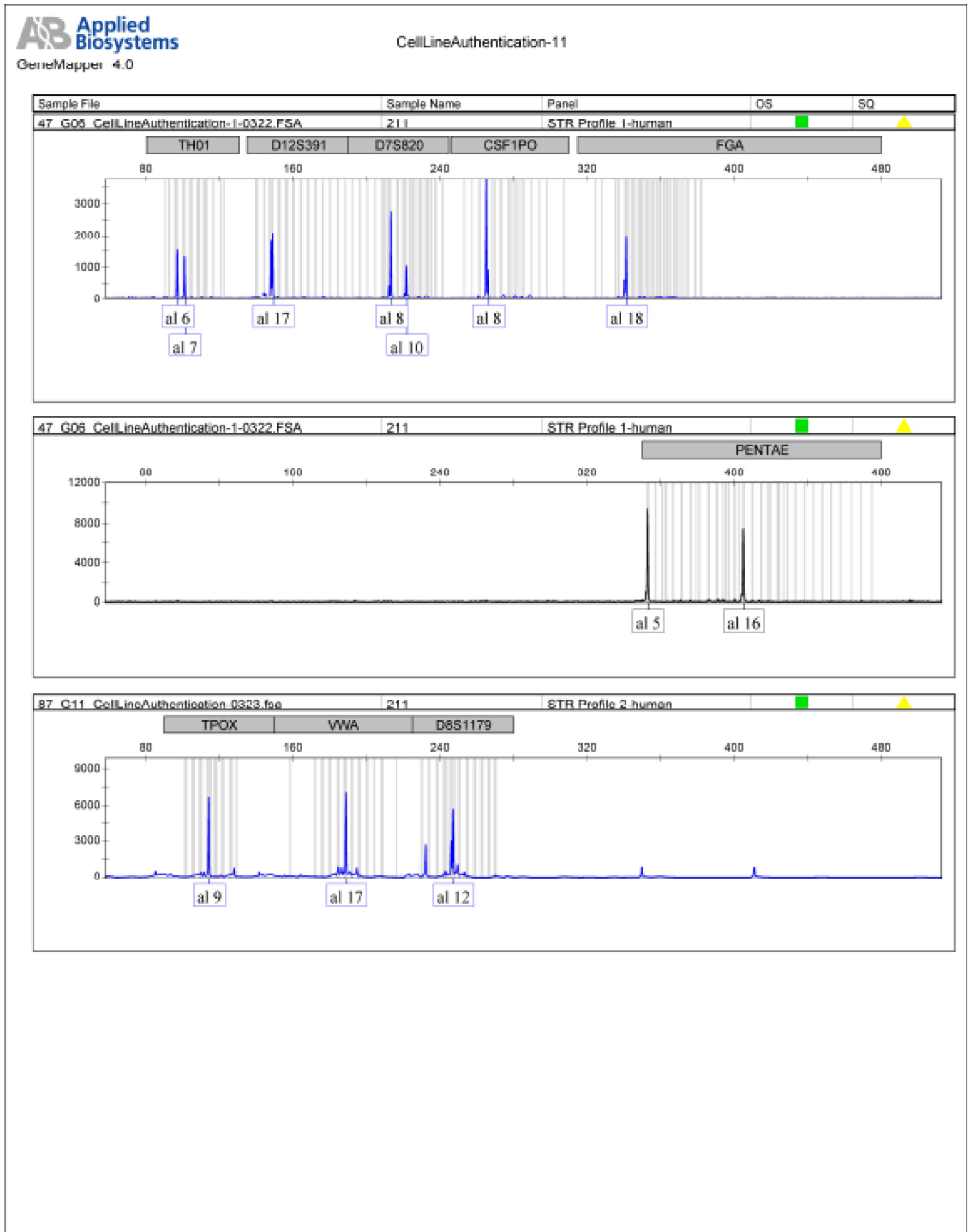
Applied Biosystems
GeneMapper 4.0
CellLineAuthentication-11
Sample File
Sample Name
Panel
OS
SQ
47_G06_CellLineAuthentication-1-0322.FSA
211
STR Profile 1-human
TH01
D12S391
D7S820
CSF1PO
FGA
al 6
al 7
al 17
al 8
al 10
al 8
al 18
PENTAE
al 5
al 16
STR Profile 2-human
TPOX
VWA
D8S1179
al 9
al 17
al 12

Applied Biosystems
GeneMapper 4.0

CellLineAuthentication-11

Sample File	Sample Name	Panel	OS	SQ
87_G11_CellLineAuthentication-0323.fsa	211	STR Profile 2-human		

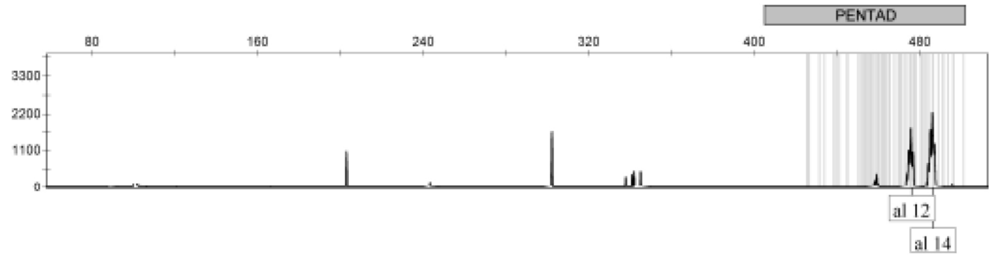

63_G08_CellLineAuthentication-0323.fsa	211	STR Profile 3-human		

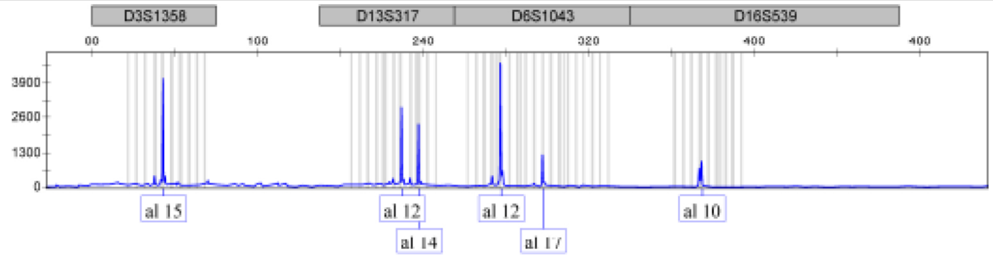

63_G08_CellLineAuthentication-0323.fsa	211	STR Profile 3-human		

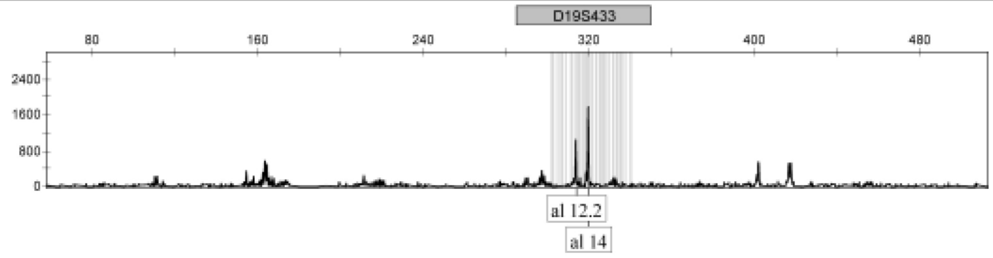

Printed by: gm

Applied Biosystems

GeneMapper 4.0

CellLineAuthentication-11

Sample File	Sample Name	Panel	OS	SQ
47_G06_XBJD1-9WZC0316(81)(1-3)1012-0324.fsa	211	STR Profile 4-human		

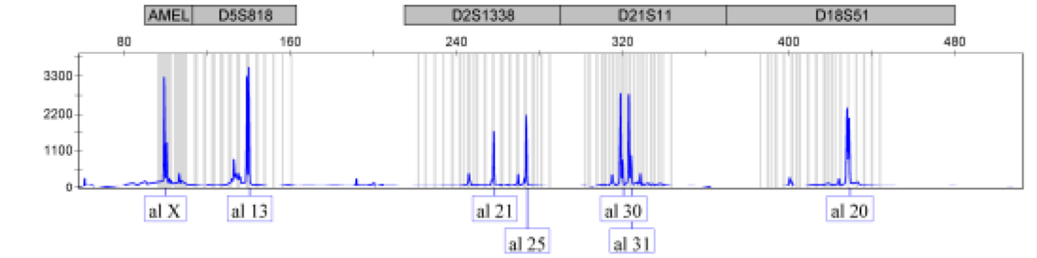

47_G06_XBJD1-9WZC0316(81)(1-3)1012-0324.fsa	211	STR Profile 4-human		

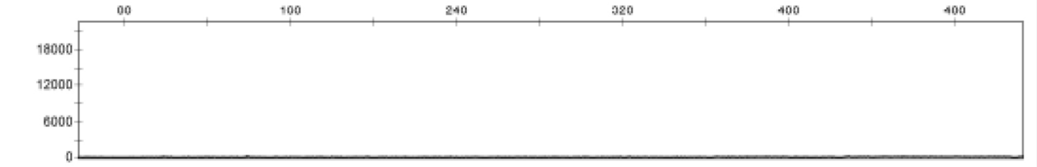

Supplement: Supplementary file 3 — cell identification reports of Hep3B [file 41419_2020_3276_MOESM3_ESM.pdf]
