# Supplementary material for: CircRNA hsa_circRNA_104348 promotes hepatocellular carcinoma progression through modulating miR-187-3p/RTKN2 axis and activating Wnt/β-catenin pathway
Source: Cell Death Dis. 2020 Dec 14;11(12):1065. doi: 10.1038/s41419-020-03276-1 (PMC7734058; doi:10.1038/s41419-020-03276-1)

细胞遗传质量鉴定检测

Cell Line Authentication Service

STR 基因型检测报告

样 品 信 息

样品编号：

客户样本编号	公司编号
HUH-7	20200601-01

样品数量： 1

样品性状： 细胞系

检测项目： STR

检测方法：用 Axygen 的基因组抽提试剂盒提取 DNA，采用 21- STR 扩增方案扩增，在 ABI 3730XL 型遗传分析仪上对 STR 位点和性别基因 Amelogenin 进行检测。

检 测 结 果

(一) 检验基本情况

公司编号	多等位基因	匹配细胞系	细胞库	EV 值	匹配说明
20200601-01	无	HUH-7	DSMZ	1.0	完全匹配

样本基因型检验结果

- 多等位基因指三等位及以上基因现象。
- 本次检测各细胞分型结果良好。

(二) 各样本描述

- 20200601-01：该株细胞 DNA 分型在细胞系检索中找到**完全匹配**的细胞系，DSMZ 数据库显示细胞名为 **HUH-7**，细胞号对应 **JCRB0304**。本次检测在该细胞系中**没有发现多等位基因**。

备注：待测细胞系与收录于 ATCC, DSMZ, JCRB 和 RIKEN 数据库的细胞系 STR 数据进行比对，未收录于以上细胞库的细胞系将无法匹配。

（三）样本分型结果

细胞的 STR 位点和 Amelogenin 位点的基因分型结果						
Loci	送检细胞 STR 信息			细胞库细胞 STR 信息		
	送检细胞名：HUH-7			细胞库细胞名：HUH-7		
	Allele1	Allele2	Allele3	Allele1	Allele2	Allele3
D5S818	12	12		12	12	
D13S317	10	11		10	11	
D7S820	11	11		11	11	

D16S539	10	10		10	10	
VWA	16	18		16	18	
TH01	7	7		7	7	
AMEL	X	Y		X	Y	
TPOX	8	11		8	11	
CSF1PO	11	11		11	11	
D12S391	20	21				
FGA	22	24				
D2S1338	19	19				
D21S11	30	30				
D18S51	15	15				
D8S1179	14	15				
D3S1358	15	15				
D6S1043	13	15				
PENTAE	11	11				
D19S433	13	14				
PENTAD	12	12				

其 他 说 明

(一) 分型方案及位点分布

	方案 1	方案 2	方案 3	方案 4
1	D3S1358	D8S1179	D19S433	AMEL
2	VWA	D21S11	TH01	D1S1656
3	D7S820	D16S539	D13S317	D5S818
4	CSF1PO	D2S1338	TPOX	D12S391
5	PENTAE	PENTAD	D18S51	FGA
6			D6S1043	

(二)　STR 数据库比对

本公司采用 DSMZ tools 进行细胞系比对，其中包含来自于 ATCC, DSMZ, JCRB 和 RIKEN 数据库的 2455 个细胞系 STR 数据。如果待检测细胞未收录于以上细胞库或这是自行建立的新细胞系将无法进行比对，用户需根据细胞分型结果自行与其他数据库进行比对。

2020-06-08

Sample File	Sample Name	Panel	SQO	OS	SQ
17_A03_CellLineAuthentication-1-0604.2.fsa	HUH-7	21Plex_STR_Panel_v0.4			

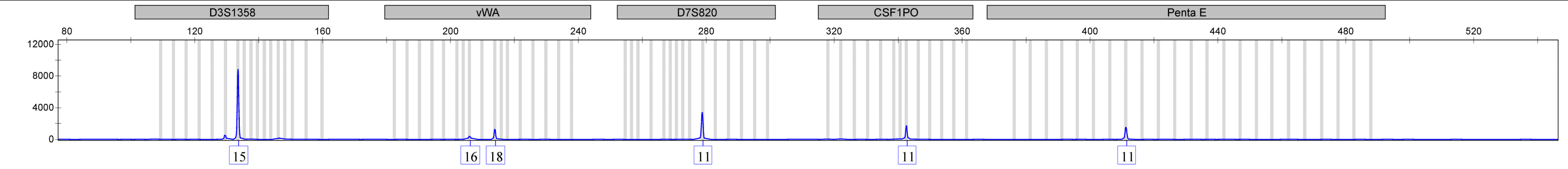

17_A03_CellLineAuthentication-1-0604.2.fsa	HUH-7	21Plex_STR_Panel_v0.4			

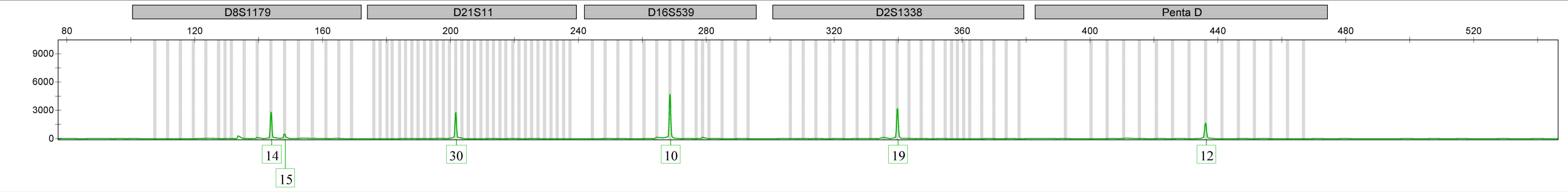

17_A03_CellLineAuthentication-1-0604.2.fsa	HUH-7	21Plex_STR_Panel_v0.4			

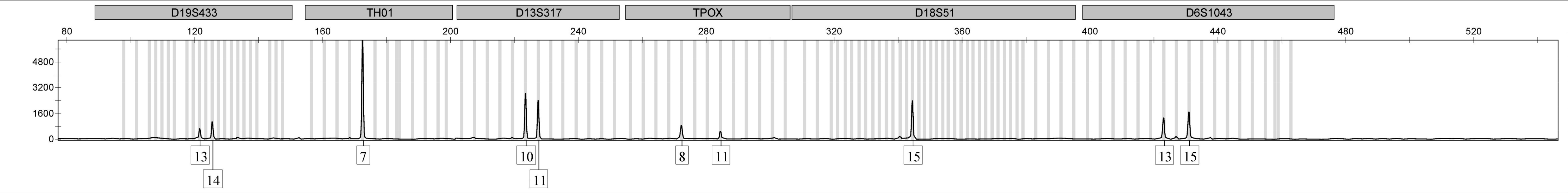

17_A03_CellLineAuthentication-1-0604.2.fsa	HUH-7	21Plex_STR_Panel_v0.4			

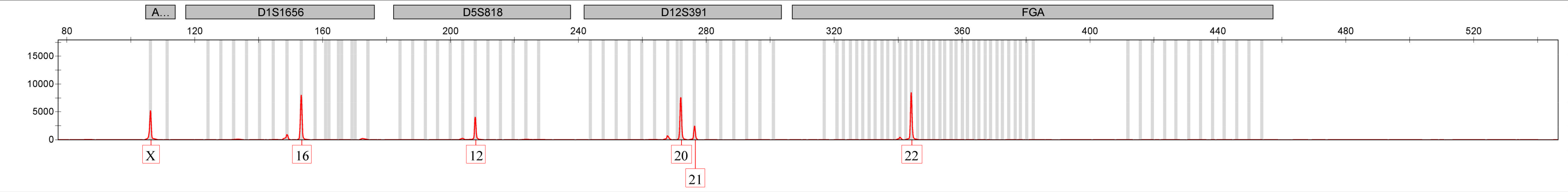

Supplement: Supplementary file 4 — cell identification reports of HUH-7 [file 41419_2020_3276_MOESM4_ESM.pdf]
